# Semimembranosus muscle displacement is associated with movement of the superficial fascia: An in vivo ultrasound investigation

**DOI:** 10.1111/joa.13283

**Published:** 2020-08-13

**Authors:** Jan Wilke, Sarah Tenberg

**Affiliations:** ^1^ Department of Sports Medicine Goethe University Frankfurt Frankfurt am Main Germany

**Keywords:** connective tissue, deep fascia, force transmission, myofascial

## Abstract

The deep fascia enveloping the skeletal muscle has been shown to contribute to the mechanics of the locomotor system. However, less is known about the role of the superficial fascia (SF). This study aimed to describe the potential interaction between the Hamstring muscles and the SF. Local movement of the dorsal thigh's soft tissue was imposed making use of myofascial force transmission effects across the knee joint: In eleven healthy individuals (26.8 ± 4.3 years, six males), an isokinetic dynamometer moved the ankle into maximal passive dorsal extension (knee extended). Due to the morphological continuity between the gastrocnemius and the Hamstrings, stretching the calf led to soft tissue displacements in the dorsal thigh. Ultrasound recordings were made to dynamically visualize (a) the semimembranosus muscle and (b) the superficial fascia. Differences in and associations between horizontal movement amplitudes of the two structures, quantified via cross‐correlation analyses, were calculated by means of the Mann–Whitney *U* test and Kendal's tau test, respectively. Mean horizontal movement was significantly higher in the muscle (5.70 mm) than in the SF (0.72 mm, *p* < 0.001, *r* = 0.82). However, a strong correlation between the tissue displacements in both locations was detected (*p* < 0.001, *r* = 0.91). A Direct mechanical relationship may exist between the SF and the skeletal muscle. Deep pathologies or altered muscle stiffness could thus have long‐term consequences for rather superficial structures and vice versa.

## Graphical abstract

Local movement of a muscle leads to mutual interaction with the superficial fascia. Both tissues are thus mechanically inter‐dependent.

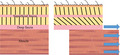

## 1 INTRODUCTION

**FIGURE 1:**
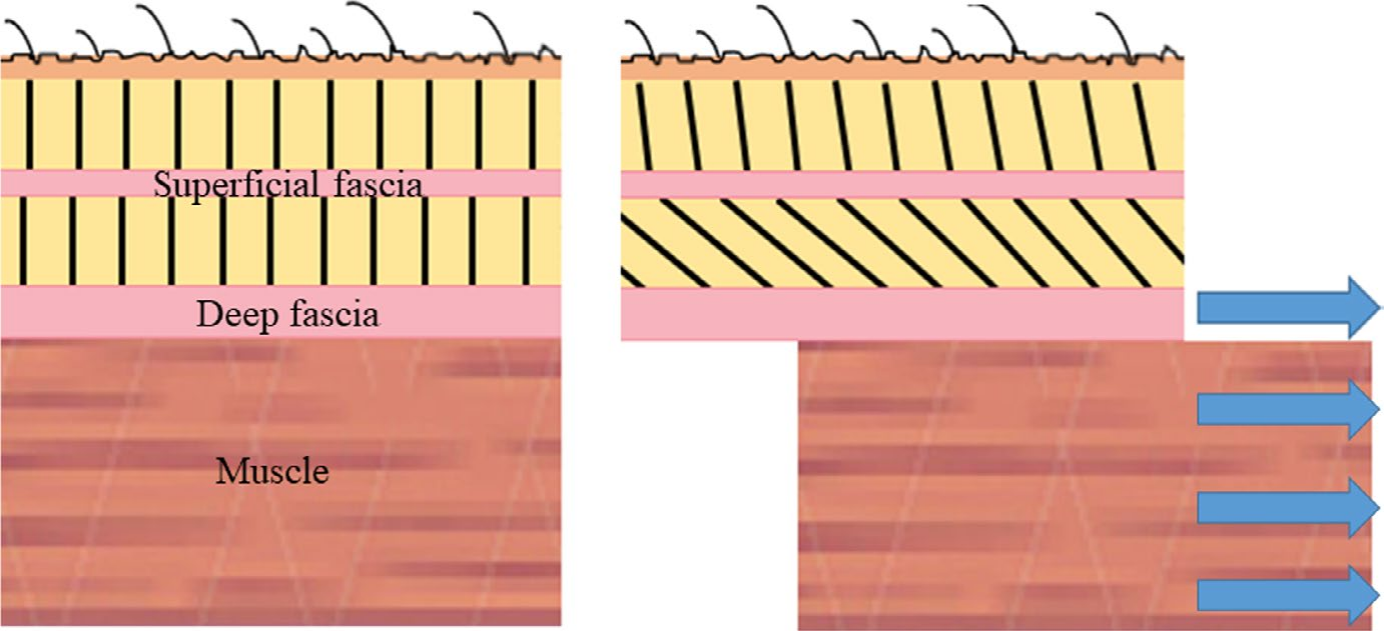
Schematic drawing displaying the hypothesized mechanical interaction between the deep fascia and the skeletal muscle. When compared to resting conditions (left), a displacement of the muscle (right) will stretch and stiffen the fibrous connections (black lines) to the superficial fascia, potentially leading to its displacement.

The deep fascia (DF) of the skeletal muscle represents a popular object of interest for scientists and practitioners in health‐related disciplines. This is mainly due to intriguing discoveries suggesting its implication in mechanical force transmission and somatic pain generation (Gibson *et al*. 2009; Yucesoy 2010; Krause *et al*. 2016; Wilke *et al*. 2017, 2018; Maas 2019). Building on the new knowledge, a variety of movement‐based, tool‐assisted, and manual therapeutic approaches targeting the fascia have been proposed (Schleip and Müller 2013; Ajimsha *et al*. 2015; Wilke *et al*. 2019). However, recently, besides the deep fascia, also the superficial fascia (SF) has received focussed attention. Being equipped with Pacini and Rufini corpuscles as well as free nerve endings, it is suggested to contribute to proprioception (Stecco *et al*. 2016). The SF has been defined as the thin collagenous layer encapsulated in the subcutaneous adipose tissue, which can be identified during dissection and in vivo using ultrasound (Lancerotto *et al*. 2011; Stecco *et al*. 2016). Morphologically, the SF attaches superiorly to the skin and inferiorly to the DF by means of superficial and deep fibrous retinacula cutis (Stecco *et al*. 2011). It may thus be speculated that motions of the muscle could affect the SF and vice versa (Figure 1). However, despite the existence of a structural linkage to the muscle, the mechanical role of the SF had been denoted as being rather small (Stecco *et al*. 2011).

The earlier assumptions regarding the limited mechanical impact of the SF have been challenged by an experiment conducted by Pamuk and Yucesoy (2015). After applying elastic Kinesio tape over the skin of the ventral lower limb, the authors used magnetic resonance imaging to observe signs of potential interactions with underlying tissues. Surprisingly, deformations did not only occur in the subcutaneous tissue but also in deeper structures such as the tibialis anterior muscle and even in non‐targeted muscles such as the toe extensors, peroneals, and the calf. This finding indeed suggests a mechanical force transmission effect between the subcutaneous and deep muscle tissue.

In view of the conflicting reports about the mechanical interaction between the SF and the DF, the objective of the present study was to gain further insight into how both tissues may transmit strains between each other. Specifically, it was investigated whether movement of a muscle and its surrounding fascia would affect the SF. We hypothesized that a displacement of the Hamstrings would stiffen its fibrous connections to the subcutaneous tissue and thus lead to a co‐directional movement of the SF.

## 2 METHODS

### 2.1 Ethical standard

The study represents a secondary analysis of data from a larger trial examining mechanical force transmission in the lower limb (Wilke *et al*. 2020). The experimental protocol was approved by the local ethics committee and the investigation was conducted according to the Declaration of Helsinki as well as the guidelines of Good Clinical Practice. All enrolled participants provided written informed consent.

### 2.2 Sample

Data from eleven healthy active individuals (26.8 ± 4.3 years, 6♂, 5♀) were examined. Exclusion criteria for study participation comprised severe orthopedic, cardiovascular, neurological, endocrine and psychiatric diseases, acute inflammatory processes or surgery in the lower limb, drug intake, muscle soreness and pregnancy or nursing period. Recruitment was performed by means of personal addressing.

### 2.3 Experimental approach

**FIGURE 2:**
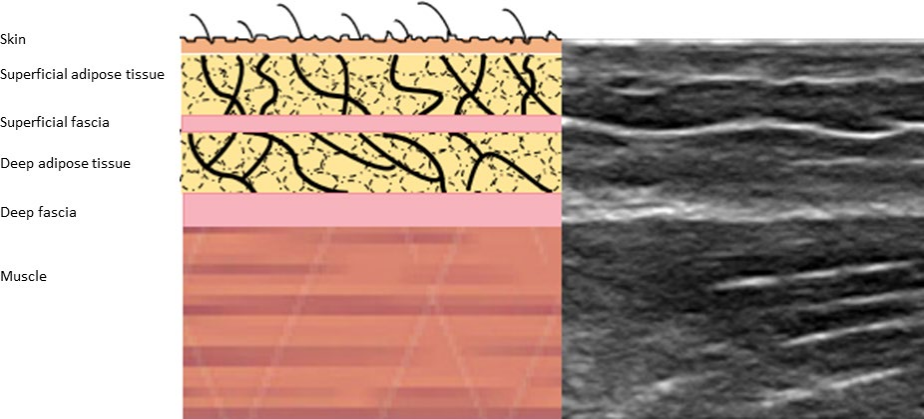
Depiction of the structural layers from the skin to the skeletal muscle as seen in the ultrasound image.

**FIGURE 3:**
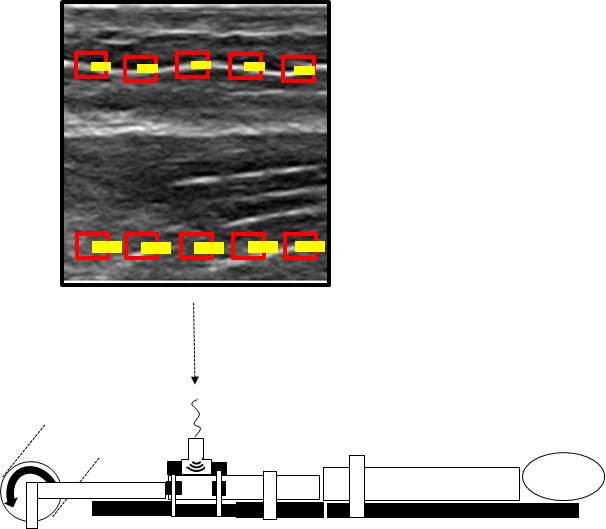
For the experiment performed in the original study, participants were positioned prone with the knees extended. The foot was passively moved into dorsal extension by an isokinetic dynamometer (curved arrow on bottom left), thereby stretching the gastrocnemius muscle. Thanks to its fascial continuity to the Hamstrings, strain can be transmitted to the dorsal thigh. Resulting tissue displacement of the semimembranosus was visualized using high‐resolution ultrasound (image in top square). In the present study, regions of interest (red boxes) were selected equidistantly in the ultrasound image (top): five in the muscle (lower boxes) and five the superficial fascia (upper boxes). A cross‐correlation frame‐by‐frame analysis served quantify the displacement of the pixels in the ultrasound image (yellow lines)

The analyzed data were collected in a trial showing that passive ankle dorsal extension induces a caudal displacement of the semimembranosus muscle (Wilke *et al*. 2020). The mechanism behind this transfer effect is assumed to be a stretch of the gastrocnemius and with this, a stiffening of a tissue continuity between the gastrocnemius and the semimembranosus. In the original study, ultrasound (US) recordings were used to visualize the displacement of the implicated muscles because it allows a clear differentiation of the various tissue layers (e.g., muscle, deep fascia, and superficial fascia; Figure 2) A schematic depiction of the previous experiment is provided in Figure 3.

In the present study, the US data visualizing the resulting movement of the semimembranosus (five consecutive repetitions) were used. The recordings of the muscle were made with a high‐resolution US device (My Lab 70, Esaote Biomedica). A linear array transducer (custom made, 100 mm, 7.5 Hz) was positioned directly over the muscle belly. To prevent artifacts induced by variations in pressure to the skin, a custom made template consisting of thermoplastic polymer was used for fixation (Cruz‐Montecinos *et al*. 2015). To detect potential probe movement over the skin, acoustically reflective markers (thin stripes of micropore tape) which are clearly visible in the US image, were placed on the skin (Morse *et al*. 2008).

### 2.4 Outcome

The maximal horizontal displacements of the semimembranosus muscle tissue as well as of the superficial fascia [mm relative to the resting position] represented the outcomes of interest. They were quantified using a frame‐by‐frame cross‐correlation analysis of the US videos. The employed algorithm, created in MATLAB (The MathWorks, Inc), was developed by Dilley *et al*. (2001) and has been shown to represent a highly reliable method to quantify tissue displacement (ICC 0.7–0.99). Briefly, the software calculates the correlation coefficient between the pixel gray levels of successive frames within previously defined, rectangle‐shaped regions of interest (ROI) of the successive frames. The pixel shift revealing the highest coefficient represents the relative movement between two frames. In the videos recorded in this trial, five equidistant ROIs were placed within (a) the semimembranosus muscle and (b) the superficial fascia above. To allow correction for potential probe movements, motion of the black zone produced by the micropore tape was measured. Mean maximal horizontal displacements of the semimembranosus and SF were calculated subtracting potential probe movement from the original values. Excellent reliability of the cross‐correlation approach for quantifying fascia displacement in the lower limb has been demonstrated in previous trials (Krause *et al*. 2019; Wilke *et al*. 2020).

### 2.5 Data processing and statistics

The tissue displacements (mm) of the semimembranosus and the superficial fascia were averaged for the five repetitions. Significant differences between both tissues' movement amplitudes were detected using the Mann–Whitney *U* test. The resulting effect size was calculated as *r* = *Z*/√*n* and interpreted according to Rosenthal (1991) as small (*r* = 0.1), medium (0.3), large (0.5), or very large (>0.8). To elucidate as to whether semimembranosus and superficial fascia displacements were systematically associated, Kendal's tau correlation was computed. According to Evans (1996), the coefficient was graded as poor (<0.2), weak (0.2–0.4), moderate (0.4–0.6), strong (0.6–0.8), or optimal (>0.8). Calculations were made with BiAS for Windows 11.2 (Goethe University, Germany); the significance level for all analyses was set to α = 0.05.

## 3 RESULTS

**FIGURE 4:**
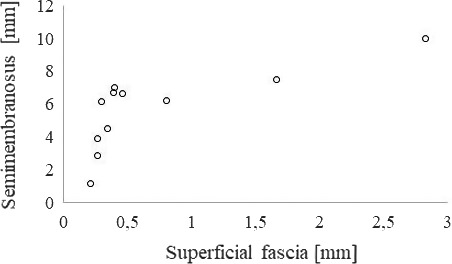
Scatter plot showing horizontal tissue displacements of the superficial fascia and the semimembranosus muscle in all examined participants

Mean horizontal displacement, corrected for probe motion which was negligible, was 5.70 ± 2.43 mm (95% CI: 4.06–7.32) in the muscle and 0.72 ± 0.81 mm (95% CI: 0.17–1.27) in the superficial fascia (Figure 4). According to the Mann–Whitney *U* test, the difference between both regions was significant and had a very large effect size (*p* < 0.001, *r* = 0.82) Correlation analysis revealed a strong correlation between muscle displacement and movement of the superficial fascia (tau‐b = .91, *p* < 0.001).

## 4 DISCUSSION

The results from the present trial raise tentative doubts about the claim that the skeletal muscles and the SF can be considered as fully independent structures. According to the analyzed data, about 13% of the induced semimembranosus muscle displacement could be tracked in the collagenous tissue layer encapsulated in the subcutaneous fat. In contrast to this, no movement was detected in the skin which confirms that the registered SF displacements were not the result of an artifact induced by probe movement.

In our experiment, the dorsal extension of the ankle stretched the calf muscles and with this, the fascial bands connecting the gastrocnemius and the semimembranosus. As these bands do not attach to the superficial fascia of the dorsal thigh, the measured SF movement seems to stem from a mutual interaction with the semimembranosus. We assume that muscle movement, via the fibrous retinacula in the deep adipose tissue, leads to a horizontally directed force acting on the SF, which could explain its displacement. This argumentation is supported by data showing that the SF, in the same way as the deep fascia, exhibits a higher stiffness in the longitudinal direction (Ruiz‐Alejos *et al*. 2016). Regarding our experimental approach, it needs to be acknowledged that the gastrocnemius connects to the entire Hamstring group (Wilke *et al*. 2016a). Although we examined the subcutaneous tissue overlying the semimembranosus, displacements of the neighboring semitendinosus may also have contributed to the SF displacement, which however, does not invalidate our general conclusion that the SF is mechanically affected by the below skeletal muscles. Besides including both medial Hamstring muscles, an intriguing perspective for future studies may be to investigate the impact of tissue gliding between the muscle and the SF on the magnitude of transmitted movement. Previous research has shown that hyaluronic acid is located between the fascial layers, functioning as a lubricant (Stecco *et al*. 2018). Variations in the amount and viscosity of the fluid could have an impact on the mechanical interaction between different tissue layers and may explain variations in transmitted forces.

Our finding of an apparent mechanical relation between deep myofascial tissue and the SF is partly in line with the study of Pamuk and Yucesoy (2015). Nonetheless, the extent of the possible interaction was not as impressive in our study. While this could be due to different experimental set‐ups (static tape application vs. dynamic stretch), it may also suggest that force transmission between both structures is direction‐specific (being larger from superficial to deep but smaller from deep to superficial) and warrants further investigation. However, it should also be noted that the thickness of the SF is highly variable throughout the body, being highest in the leg (Abu‐Hijleh *et al*. 2006). Consequently, as both, the study of Pamuc & Yucesoy and our trial were conducted targeting the lower limb, the observed mechanical interactions may not occur or be of lower magnitude in other locations.

The mechanical reciprocity between the DF and the SF could be of relevance for therapists and coaches. Firstly, it substantiates the assumption that manual treatments over the skin can affect deeper structures not only by means of compression but also by the application of shearing forces. It further suggests that, vice versa, movement or abnormalities in deep structures (e.g., increased muscle stiffness) could have consequences for the SF. Finally, it corroborates the recommendation for manual treatments to use only light pressure applied to a large surface when aiming to treat the SF in an isolated manner (Stecco *et al*. 2016). While these implications primarily relate to the work of practitioners, researchers should note that the subcutaneous tissue cannot be used as a reference when aiming to calculate soft tissue displacement of tendons, muscles, or their associated fasciae: As deep movement is highly correlated with superficial displacement, choosing this approach would induce a strong bias.

Some methodological considerations have to be made. The investigation was performed under passive conditions without voluntary muscle activity. Possibly, force transmission would differ markedly in ex‐laboratory situations. Synergist and antagonist contractions can be expected to substantially limit or enhance the effects, particularly because they are morphologically linked and may mutually interact with each other (Huijing 2003; Maas and Sandercock 2010; Yucesoy 2010; Maas 2019). In the same vein, the speed and amplitude of semimembranosus displacement need to be considered which, due to the controlled experimental setting, had to be very slow and small. Fast movements with large muscle elongations, for example, in sports, could provoke very different interactions with the SF. Another issue relates to the induction of semimembranosus displacement. It was produced using a remote exercise effect stemming from movement of the ankle joint. While the muscle motion in the semimembranosus was most likely resulting from a force transmission through a fascial band connecting it to the gastrocnemius, it has to be acknowledged that there is also a connection between the crural fascia and the fascia lata (Stecco *et al*. 2008; Wilke *et al*. 2016b). It is, therefore, possible that the SF movement resulted from both, a serial force transmission between them and the interaction of the SF and the underlying deep tissue. However, in view of the strong correlation between muscle and SF displacement, we consider the likelihood that the motion was due to an superior‐inferior interaction (SF and semimembranosus) by far higher than that stemming from a caudal‐cranial (crural fascia, fascia lata, SF of semimembranosus) interaction. Yet, it would thus be intriguing to conduct a similar study only provoking local movements without non‐local force transmission to further investigate this issue. While this is not possible with elongation (directly stretching the Hamstrings would also stretch their SF), a contraction of the muscle could be elicited.

## 5 CONCLUSIONS

The structural linkage between deep myofascial tissues and the superficial fascia seems to be capable of transmitting small but relevant forces. Researchers and practitioners should be aware of this finding when designing clinical trials or tailoring therapeutic (e.g., manual) treatments.

## ACKNOWLEDGEMNTS

Open access funding enabled and organized by Project DEAL.

## CONFLICT OF INTEREST

None reported.

## Data Availability

The data will not be shared.
